# The Role of Nutraceuticals in Chemoprevention and Chemotherapy and Their Clinical Outcomes

**DOI:** 10.1155/2012/192464

**Published:** 2011-12-07

**Authors:** Sabita N. Saldanha, Trygve O. Tollefsbol

**Affiliations:** ^1^Department of Biology, University of Alabama at Birmingham, 175A Campbell Hall, 1300 University Blvd, Birmingham, AL 35294-1170, USA; ^2^Department of Math and Sciences, Alabama State University, P.O. Box 271, Montgomery, AL 36101-0271, USA; ^3^Clinical Nutrition Research Center, 402 Webb Nutrition Sciences Building, 1675 University Blvd, University of Alabama at Birmingham, Birmingham, AL 35294-3360, USA; ^4^Comprehensive Cancer Center, University of Alabama at Birmingham, 1802 6th Avenue South, North Pavilion 2500, Birmingham, AL 35294, USA; ^5^Center for Aging, University of Alabama at Birmingham, 933 South 19th Street, Room 201, Community Health Services Building, Birmingham, AL 35294-2041, USA; ^6^Nutrition Birmingham Obesity Research Center, University of Alabama at Birmingham, 402 Webb Nutrition Sciences Building, 1675 University Blvd, Birmingham, AL 35294-3360, USA

## Abstract

The genesis of cancer is often a slow process and the risk of developing cancer increases with age. Altering a diet that includes consumption of beneficial phytochemicals can influence the balance and availability of dietary chemopreventive agents. In chemopreventive approaches, foods containing chemicals that have anticancer properties can be supplemented in diets to prevent precancerous lesions from occurring. This necessitates further understanding of how phytochemicals can potently maintain healthy cells. Fortunately there is a plethora of plant-based phytochemicals although few of them are well studied in terms of their application as cancer chemopreventive and therapeutic agents. In this analysis we will examine phytochemicals that have strong chemopreventive and therapeutic properties *in vitro* as well as the design and modification of these bioactive compounds for preclinical and clinical applications. The increasing potential of combinational approaches using more than one bioactive dietary compound in chemoprevention or cancer therapy will also be evaluated. Many novel approaches to cancer prevention are on the horizon, several of which are showing great promise in saving lives in a cost-effective manner.

## 1. Introduction

The transformation of a normal cell into a cancerous phenotype requires stages of initiation, progression, and promotion by altering specific genes [1–3]. Although predisposition to cancer cannot be signaled out by a single factor, a group of factors place some individuals at a higher risk of acquiring the disease. Most of the high-risk cases may have a genetic background, but in some instances dietary choices can dictate the outcome of health. As determined by population and epidemiological studies, the predominant forms of cancer and cancer-related deaths are those of the lung and bronchus, breast, colorectal, and prostate [4, 5]. These cancers are also more prevalent in the western parts of the world and are much lower in Asian countries. A well-balanced diet that includes more of vegetables and fruits with less fat/meat intake is in most cases a staple of many Asian countries [4, 5]. Many hypotheses have supported that diet and environment greatly influence cellular function and health [6]. 

**Figure 1:**
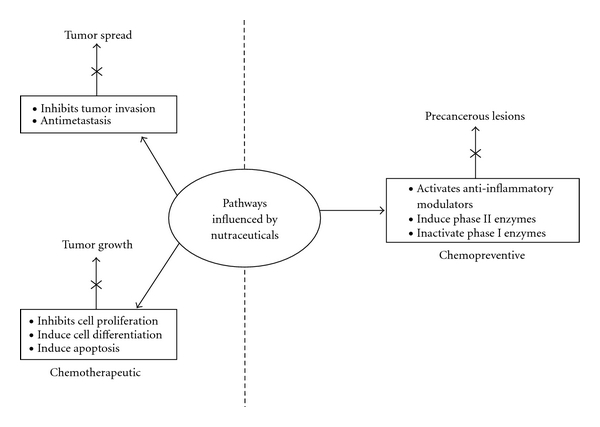
Cellular pathways affected by the activities of bioactive components in dietary sources. Of the natural compounds present in dietary sources, some are more involved in regulating chemopreventive pathways and some are more effective in influencing chemotherapeutic pathways. However, a few of the bioactive molecules found to date can impart both chemopreventive and therapeutic effects, such as EGCG and genistein. Compound combinations as discussed in the paper that can affect different pathways are shown and can have profound effects on tumor growth and inhibition.

Phytochemicals are plant-based chemicals that mediate their positive health benefits directly, by affecting specific molecular targets such as genes, or indirectly as stabilized conjugates affecting metabolic pathways [7]. Many genes play significant roles in the cell cycle pathway, and some of these are altered in cancer cells [1, 2]. The aim of most studies is to understand and formulate mechanistic pathways by which these naturally derived chemicals can alter the fate of a cell. For a cancerous cell to survive, it should be able to proliferate, obtain energy, and establish angiogenic pathways, in a tumor mass. Altering genes that affect these pathways can serve as suitable tools to decrease tumor mass and also allow for tumor regression. In this paper, the key focus will be on mechanistic pathways that are regulated by nutraceuticals to bring about changes in the tumor environment and serve as alternative approaches for cancer prevention and therapy (Figure 1). 

The study of phytochemicals and the classification of these compounds have been previously reviewed [8]. However, in this paper only some of the most potent and promising chemopreventive and therapeutic molecules will be analyzed, with emphasis on combination therapy of these with other nutramolecules. Most phytochemicals derived from dietary sources are classified under an umbrella of specific chemical compounds as detailed in Table 1. These molecules may not have a nutrient value but are germane to the function of a cell. Various studies have shown that these molecules can induce apoptosis, inhibit cellular proliferation, affect angiogenesis, and affect cancer metabolism in various cancers, all of which are hindrances to tumor growth (Figure 1) [7]. 

Several of the phytochemicals listed in Table 1 have been investigated in terms of their curative properties. However, one must carefully interpret the observed results *in vitro *and *in vivo *before testing the same in a clinical setting. The reasons for this are manyfold. Tests in culture are pure, in that there is only one cell type in the culture plate and all conditions are controlled, including the bioactive compound. *In vivo, *however, the scenario changes as there are a host of other factors that need to be taken into account, including age, weight, diet, and metabolism of the compound. A bioactive molecule in culture may be subjected to less metabolic changes and may be presented to the cell in its native form. However, *in vivo *the same compound may be presented differently, perhaps as a conjugate, and its mode of action may change amongst the multitude of other molecules in the host's microenvironment. Many *in vivo *experiments also control for the type of diet being administered to the organism, where the concentrations or plasma availability can be adjusted. Therefore, what may work well *in vitro*, may have no agonistic effects or even antagonistic effects *in vivo, *and such discrepancies are often seen when comparing population and epidemiological studies in terms of chemical efficacy. 

An effective nutraceutical is one that will have a low nontoxic dose while creating a magnitude of change in tumor dynamics. This means that at a low dose the compound should act fast on the tumor load. However, if the time taken to be effective is slow, the problems faced would be maintaining a tolerable dose and increasing bioavailability and stability. A solution to such a problem would be to use a combinatorial approach to therapy, a bioactive molecule with an effective synthetic drug or double-nutratherapy (e.g., curcumin and resveratrol). Once tumor regression sets in, dietary composition of the molecule can be adjusted.

## 2. Nutraceuticals and Their Preventive and Therapeutic Roles

### 2.1. Genistein: A Potent Isoflavone

Many phytochemicals are currently being investigated for their promising anticarcinogenic properties. *In vitro *investigations have shown that some compounds exert their antitumor functions at much higher concentrations and that dietary consumption is insufficient to achieve such effective concentrations at the tumor site. Therefore, the mode of delivery is a very important factor that needs to be considered at clinical trials and during *in vivo *studies. The nontoxic properties of natural compounds are essential to the design of a formulated therapy. However, evidence along several lines of treatment has shown that some compounds are preferentially more potent in activity when administered early in life [9, 10]. For instance, soy-based prevention of breast cancer is thought to be more successful when soy products and their derivatives are consumed in early development [9]. 

Isoflavones are a group of phytochemicals that are predominant constituents of a soy-based diet [9, 10]. Among isoflavones, the three major constituents that have been shown to have remarkable influences in cancer prevention and therapy are genistein, diadzein, and glycitin [11]. They are collectively grouped as phytoestrogens for their weak estrogen-like activity and bind preferentially to ER-*β* receptors [12–15]. Evidence of antiproliferative activity of genistein *in vitro *stems from its ability to inhibit the tyrosine kinase enzyme that is most often upregulated in cancer cells [16, 17]. As a chemopreventive agent, genistein is thought to influence the differentiation process of mammary tissue. It is believed that early differentiation of mammary tissue into terminal buds, as seen in rats, serves as a chemopreventive strategy as it reduces the susceptibility of the epithelial cells in the ducts to carcinogens or estrogen and the ontogeny process [9]. Many aggressive cancers have altered epidermal growth factor (EGF) receptors on their cell surface allowing for a continuous downstream signaling pathway for cell division [18, 19]. This is interesting, as genistein can serve as a two-fold approach molecule for prevention and treatment. When EGF binds to its receptors, tyrosine kinase activation results in the phoshorylation of tyrosine residues of proteins involved in downstream cell signaling pathways that trigger cell division. Though studies have shown that genistein increases the EGF transcript early in development of mammary tissue, this perhaps is essential for differentiation and faster development of the breast tissue. In the long run this is a positive preventive strategy of breast lesion formation in ducts [9]. However, as seen in older rats [9], EGF mRNA decreases. Therefore, a decrease in EGF mRNA coupled with inhibition of tyrosine kinase by genistein would profoundly decrease tumor growth as cell signaling pathways are crucial to tumor maintenance.

Numerous studies have highlighted the antiproliferative role of genistein in various cancers; however, there are some studies indicating that genistein may increase cell proliferation [19, 47]. A key point to note is that nutraceuticals can be effective based on the form of genistein or its dose given at the time of the study (Tables 2 and 3), especially with respect to *in vitro *and *in vivo *models. Importantly, the downstream targets of bioactive molecules under investigation need to be ascertained for each specific tissue, if overall health applications are an issue. The nutraceutical may not affect a specific common pathway for tumors of different origins. For example, in breast tissue, EGF may be highly expressed, but, in colon cells or pancreatic cells, genes that regulate cell division other than EGF may be affected [48]. Cell culture experiments using plant-based nutrients depend on the sensitivity of the cells that are being investigated. When cell lines are established, they are derived from cancerous tissues of specific organs and are, therefore, cell-type specific. This is drastically different in a clinical setting where the molecule has to mediate its activity amongst a host of various molecules and cell types. Therefore, the concentration of the phytonutrient in the supplemented diet will be crucial to its efficacy in the tumor environment. This can help explain the discrepancies seen in clinical trials of genistein for different tissues [47, 49, 50]. Outcomes of some *in vitro* studies suggest that, like other bioactive compounds, genistein appears to have a specific cut-off concentration at which this isoflavone can exhibit anticarcinogenic activity (10 *μ*M or even higher) [48, 51], and it is, therefore, imperative to achieve such concentrations *in vivo*.

 Isoflavones, in particular, genistein, have been extensively studied as prospective antitumor molecules in the treatment of prostate cancer [19, 52, 53]. There has been a well established line of evidence that genistein works against prostate cancer, but a majority of studies are *in vitro *in cultured cells [19, 52–56]. Limited clinical trials have tested the therapeutic efficacy of genistein in prostate cancer and those that have revealed inconsistencies in cell proliferation and tumor growth [57–60]. Given the inconsistencies in some of the outcomes, emphasis should be on the dose of the supplement and the form of the nutrient in the supplement at the time of administration to the patient in clinical trials. The highest achievable plasma concentration of isoflavones is 1 *μ*M through orally administered food sources. From previous studies, this concentration is not sufficiently significant to bring about anticarcinogenic effects on the tissue. However, there is ample evidence that genistein and other isoflavones do exhibit anticancer properties and inhibit cell proliferation and tumor growth. A clinical study by Gardner et al. [61] showed that treatment of patients with dietary supplements (82 mg/day aglycone equivalents) of isoflavone yielded a higher concentration of total isoflavones in the prostatic tissues than in serum. Therefore, there is a possibility of increasing the concentration of isoflavones to anticarcinogenic levels in tissue.

An orally administered dose of isoflavones must withstand the rigors of the alimentary canal and become metabolized before they can be made available to tissues. Most isoflavones exist as conjugates rather than in their free state. This conjugation is perhaps the best way to present the molecule to the cell in tissues, and the hydrolysis of the conjugates in the tissue allows available free genistein delivery to the cells, as presented or tested *in vitro*. For pharmaceutical companies, it is required to formulate supplements with precise ratios of individual constituents of the compound. Unless a very pure form, a capsule or supplement may contain a mixture of genistein, diadzein, and glycetin (Tables 1, 2, 3, and 4). The percentage of each nutrient in the mixture will have a profound effect on the bioavailability of the compound after metabolism (Tables 2 and 3). To design such a product is certainly not easy and is dependent on many factors, but the two essential factors are the grade/stage of the tumor and the site or origin of the tissue. Of the two isoflavones, diadzein has been shown to have a lesser apoptotic effect on prostate cancer cells but can inhibit neoplastic transformation [61]. Therefore, it would be advantageous to use supplements containing the two bioactive nutrients as chemopreventive agents.

Of the predominant high-risk cancers, genistein appears to have a greater affect on prostate cancers [52–54]. Genistein mediates the apoptosis of cancer cells by activating and/or inhibiting genes and/or enzymes germane to tumor maintenance (Figure 1, Table 4). Some of these important mechanisms are the inhibition of the activity of tyrosine kinase, nuclear factor kappa B (NF-*κ*B), and vitamin D 24-hydroxylase [86], activation of tumor suppressor genes, and modulation of androgen-responsive gene expression, prostate-specific antigen (PSA), and the androgen receptor (Table 4). Of the prominent isoflavonones in soy, diadzein is less effective in its action on prostate cancer, but, unlike genistein, it is metabolized to equol, an isoflavandiol which has a longer half-life than genistein [87]. The longer half- life of equol creates the possibility of using this chemical in combination with other available nutraceuticals, where the net effect may be synergistic. However, prior preclinical tests are required to investigate this possibility. 

Other dietary compounds are also of great interest in this regard. *In vitro*, vitamin D (Vit D) has potent tumor prevention ability and can induce differentiation and apoptosis in some of the most predominant cancers [48]. The use of nutrients as a possible treatment approach is based on the fact that chemicals occurring naturally will minimize side effects when applied to a biosystem. However, the *in vitro *dose at which Vit D induces its antitumor properties causes hypercalcemic conditions that can preclude treatment in patients [49]. In prostate cancer, a leading cause of cancer deaths in the western parts of the world, androgen ablation therapy is the choice of treatment. However, as the cancer becomes aggressive, hormone ablation therapy fails, and progression ensues via androgen-independent pathways. Therefore, alternate therapies are very much in demand. Vitamin D is an alternate form of treatment in prostate cancer (PCA) and is shown to induce apoptosis in PCA cells *in vitro*. However, all PCA cell lines *in vitro *are not equally receptive to the vitamin D treatment or genistein [88]. Cell lines such as DU145 prostate cancer cells are especially more resistant as they express high levels of CYP24, an enzyme that catabolizes Vit D3 into less active metabolites [88]. To circumvent this problem, a recent study showed that a dual combination therapy, of DU145 to genistein and Vit D3, increased the sensitivity of the cells to Vit D3 by decreasing CYP24 expression. What is interesting to note is that the combination approach not only lowered the effective dose, but was able to abrogate cell proliferation as well. This lowered concentration of genistein at 100 nM is achieveable *in vivo *through dietary sources, and clinical studies would be required to determine the localization of genistein and Vit D3 in prostatic tissues.

An *in vivo *study for colorectal cancer has demonstrated a similar effect [89], but in this case the mice were given a single gavage of 250 *μ*g of genistein. This mode of nutrient administration is useful for a preclinical test and probably has applications as a chemopreventive supplement. However, in terms of a clinical setting, patients are often exposed to a host of other nutrients or isoflavones in their diet, and; therefore, an *in vivo *model replicating such an environment with various percentages of isoflavones will allow for a better understanding of concentration and bioavailability of genistein that can mediate an apoptotic effect and reduce CYP24 expression in colonic tissues in the presence of vitamin D. 

The antimetastatic properties of genistein are mediated by altering the expression of NF-*κ*B, and inhibiting the tyrosine kinase enzyme [17, 90]. Non-small-cell lung cancer (NSCLC) is a highly aggressive form of lung cancer with a poor prognosis. Therefore, alternate approaches that drastically reduce tumor growth are of utmost importance. Activation of epidermal growth factor receptor tyrosine kinase (EGFR-TK) enhances the cell signaling pathways allowing tumor growth. The use of drugs that inhibit EGFR-TK and affect NF-*κ*B, a gene whose transcribed products are essential for invasion and metastasis, can induce a more aggressive approach of reducing tumor size and the spread of the disease. A clinical therapy should be aimed at reducing tumor growth and spread by inhibiting mechanisms that contribute to the activation of metastasis. In NSCLC, genistein remarkably enhances the effects of EGFR-TK inhibitors, such as erlotinib and gefitinib, when used in combination with each of them, respectively. This effect was seen to be mediated by a marked reduction in NF-*κ*B and others, such as EGFR, pAkt, COX-2, and PGE(2), essential for regulating genes that control division, proliferation and metastasis [90]. A few studies have shown how a combined approach can lower the effective dose concentration even of chemotherapeutic drugs, minimizing potential side effects. A study conducted on breast and pancreatic cells showed that, when the cells were primed with genistein, lower concentrations of the chemotherapeutic drugs were needed to significantly bring about growth inhibition and apoptosis than with the drugs alone. In addition, NF-*κ*B was transcriptionally inhibited in the combined treatment [90]. 

From a number of investigations, a common thread of evidence seems to emerge that considerable variation in the efficacy of bionutrients in cancer treatment exists and differs even among the same cell lines tested. The reasons for this are manyfold (Table 5). Cell lines derived from the same tissue hypothetically should be sensitive to the same dose or chemical class of the phytonutrients, but such is not always the case. Alternate medicinal approaches have an important task to identify crucial factors that change the sensitivity of the chemical and determine chemical modifications that would be necessary to modulate more synchronized results across several cell lines expressing similar genotypic and phenotypic signatures.

### 2.2. Epigallocatechin-3-gallate (EGCG): A Potent Flavanol

Of the major food-derived phytochemical constituents that are extensively studied for their chemopreventive and chemotherapeutic use, EGCG and genistein are by far the most investigated. EGCG has been shown to have numerous anticancer properties which include antiangiogenic activity by affecting the transcriptional expression of vascular endothelial growth factor (VEGF) [91], inhibiting tumor initiation and promotion by inhibiting signal transduction pathways via [phosphatidyloinositol 3-kinase-Akt kinase- NF-*κ*B] [92–94], inhibiting EGFR [95], inhibiting Her-2 receptor phophorylation in breast carcinoma cells that constitutively expresses Her-2/neu receptor [95], inducing apoptosis in estrogen receptor-(ER-) independent breast cancer cells [96], causing antimetastatic activity [97], inhibiting proteasome formation [98], inhibiting glucose-regulated protein (GRP78) activity [99]; inhibiting insulin-like growth factor-I receptor (IGF-IR) [100], and preventing invasion of tumors by inducing HMG-box transcription factor 1 (HBP1) transcriptional repressor, an inhibitor of the Wnt signaling pathway crucial for tumor-invasive property [101]. 

The serum level concentrations of EGCG are important to ensure that an effective response is seen without adverse or even tumor-promoting functions. Studies have shown that high doses of catechins that include a higher concentration of two prominent compounds, epicatechin gallate (ECG) and EGCG, induce hypoxia-inducible factor 1 which is responsible for activating genes related to hypoxia conditions. This allows tumor cell proliferation through alternate survival pathway mechanisms [102]. Most breast cancers are ER dependent; however, for breast cancers and others that are ER independent, EGCG inhibits the growth of tumor cells through the process of apoptosis [96, 103]. As seen in MDA-MB-468 ER-negative cells, cellular apoptosis is mediated by inducing p53 and Bax proteins that enhance apoptotic functions in cells [96]. Such observations have been corroborated by *in vivo *studies using animal models [97]. 

Most studies have shown that anticancer properties of EGCG are mediated at higher doses. However, such doses may be irrelevant to clinical applications as they may be physiologically unachievable through dietary consumption. Therefore, clinical trials should be aimed at achieving desired anticancer preventive or tumor functions at much lowered doses. Such outcomes are possible with a dual-drug approach. One study [95] demonstrated that combining EGCG with the drug taxol, which is commonly used to treat breast carcinomas, lowered the effective dose of EGCG, ranging from 0.1–1.0 *μ*g/mL which is a serum obtainable level through metabolism. This same group showed that higher doses (30–40 *μ*g) of EGCG were required to mediate a similar effect when used alone [95]. 

EGCG can be exploited as a chemopreventive agent if it prevents cancerous lesions from occurring at lower dose concentrations and for prolonged periods of time. Most *in vitro *studies have used relatively high doses of EGCG and such doses may prove to be more tumor promotive than preventive in longer exposure time periods. In a study designed by Pianetti et al. [92], contradictory results on the effects of EGCG on Her-2/neu overexpressed receptor in NF639 breast cancer cells was observed. At short exposure times, EGCG was very effective in reducing cell proliferation, but at prolonged exposure cells became resistant to EGCG with increased levels of NF-*κ*B. This observed change in drug-induced resistance was related to the activation of mitogen-activated protein kinase. It appears that single doses or one specific chemical constituent is mostly insufficient to induce tumor suppression or regression. Such *in vitro *data outcomes emphasize that a dual-drug treatment approach is necessary to treat the disease. This also signals that the timing of the nutradrug that is administered is important. Perhaps EGCG should be administered early in treatment, but later other phytochemicals or drugs, in conjunction with EGCG, may need to be administered in the treatment regimen. In their dual-drug treatment of NF639 Her-2/neu breast cancer cells, Yang et al. found that treating the cells initially with EGCG lowered cell proliferation and the later introduction of the MAPK inhibitor, U0126, reduced invasive phenotype [93]. 

Most studies determining the anticancer drug properties of EGCG are preclinical. For better understanding of specific EGCG effects, clinical trials should be carefully designed to include parameters that influence EGCG effectiveness. EGCG has different roles in ER-dependent versus ER-independent receptors, and, therefore, the type of diet needed to emulate *in vitro *doses need to be clearly understood through clinical trials and careful pharmacokinetic studies of these doses in healthy individuals, ER-positive breast cancer patients, and ER-independent tumors. 

In testing phytochemicals of the same or different class it is rather uncertain which markers are necessary to determine comparable dosage values for *in vitro *versus *in vivo *efficacies. Formulation of a diet is one of the major deciding factors in the functional efficacy of a chemical constituent. It defines the concentration of the dose that will be available *in vivo*, after metabolism, and determines the diet that needs to be given to achieve such an outcome. Even though single-dose individual or mixed phytochemical treatments are currently available to cancer patients, they are relatively new and much more research in this direction is warranted. One such therapy that is rapidly gaining importance and holds promise for future cancer treatments is combination therapies using plant-based chemical compounds known as nutraceuticals.

## 3. Combinatorial Therapy: A Promise of the Future (See Table 3)

In prevention or treatment, combinatorial approaches can be of the following types: a phytonutrient and an effective drug, two or more phytonutrients, a synthetic phytonutrient and an effective drug, or a synthetic phytonutrient and a natural nutrient. Studies in the last few decades have focused attention on unraveling the protective properties and mechanistic actions of many phytochemicals. Still the pharmacokinetics of quite a few of these phytochemicals are not known, and, for a few that are known, there is much variability based on mode and form of delivery, dose, and the model organism of study (Tables 2, 3, and 4). Another interesting approach to enhancing curative and preventive properties of these nutrients is combination therapies. The therapy is based on the factual information available at hand and using the potent properties of one with that of another to enhance synergistic or additive actions (Figure 1). In this paper, groups that have worked with different phytomolecules belonging to a different or the same chemical class of compounds have been analyzed for their antitumorigenic activities, and the overall results of the experiments for each group are described in Table 4.

### 3.1. Curcumin and Taxol (See [63])

Primary breast cancer cells are commonly treated with the drug taxol. Sustained chemotherapeutic treatment with this drug has often resulted in drug resistance and tumor progression. Many chemotherapeutic drugs induce the expression of the metastatic gene NF-*κ*B which encourages tumor progression. Interestingly, natural-based compounds that are pharmacologically safe have been shown to inactivate NF-*κ*B expression. Taxol is a powerful drug in the treatment of cancer therefore, in order to prevent metastasis, a combination of Taxol with curcumin has been shown to downregulate the expression of NF-*κ*B and induce apoptosis.

### 3.2. Curcumin and Xanthorrhizol (See [64])

A study conducted on an invasive breast tumor cell line, MDA-MB-231, has shown how and when compounds added to the cells determine the overall efficacy of the treatment. A sequential addition of curcumin and xanthorrhizol (a rhizomal sesquiterpenoid of *Curcuma xanthorrhiza*) in culture resulted in additive and antagonistic effects depending on which compound was added first to the culture. However, simultaneous addition of the compounds resulted in synergistic effects at lower concentrations and agonistic effects at higher concentrations. Such experiments provide evidence that the efficacy of a drug is dependent on dose, time, and how it is presented to the cells. Therefore, results obtained might be contradictory if doses used are simply antagonist or additive. For a successful combination therapy or prevention, synergistic doses are more relevant to mediate downstream effects, as lower concentrations of the test biomolecules will be required.

### 3.3. Curcumin and Docosahexaenoic Acid (DHA) (See [65])

DHA is a dietary compound present in fish oil that has been shown to have potent chemopreventive affects against cancer. Chemotherapeutic effects of compounds are often analyzed using *in vitro *models. However, what is most often observed is that all cells from the same tissue sample do not react the same way to the test compound. It is essential to have a chemopreventive or therapeutic agent that can induce its effects on a wide range of cancerous cells arising from the same tissue. In this study, the authors analyzed five cell lines expressing different cell surface receptors (Table 6) which make them susceptible to chemotherapeutic compounds but in different ways and to different degrees. The combinatorial synergistic doses for each cell line were different, as shown in the Table 4. In particular, one breast cancer cell line, SK-BR-3, which is ER-negative exhibited a higher uptake of curcumin in the presence of DHA. DHA does not directly contribute to cell inhibition, but the combination of this compound with curcumin greatly enhances the uptake of curcumin by the cells. This compound, DHA, can reach a plasma concentration level of 200 *μ*M. Although the focus of this study was entirely based on the SK-BR-3 cell line, the effects of reduced synergy on other cell lines in terms of transcriptome effects need to be investigated. Mammary tumors may contain a heterogenous population of cells exhibiting different surface receptors. Using combination therapy should be aimed at reducing the populations of all these cell types within the tumor site to truly exhibit antitumor potency with minimal side effects.

### 3.4. Curcumin and Genistein (See [66]): A Preventive Strategy

The aim to use natural compounds in diets is to render the chemopreventive properties of the compounds to the tissues. Numerous studies have shown that single dosage of compounds used alone is effective for chemoprevention. The problem faced is the inability to achieve high serum concentrations *in vivo*. Although combination studies are just beginning to surface as more prominent approaches in clinical treatment, studies, though limited, have shown that synergistic effects of the compounds are able to be achieved at much lower doses than when compounds are used alone. Especially in cancers that are hormonally regulated, the tissues are often exposed to external or internal hormonal stimulation, like estrogen, as in the case of breast tissue. Environmental agents that mimic estrogen-like activity can often stimulate or initiate the carcinogenic process. Curcumin, a curcuminoid, and genistein, an isoflavone, are derived from two different chemical classes, yet they have been known to inhibit a variety of tumor types *in vitro *and *in vivo*. Clinical trials of these compounds individually have been tested [19, 60, 104, 105]. The mechanistic action of the individual compounds in many different cancers has been investigated as well. However, using these compounds in combination drastically affects the development of tumors by mediating changes in shape and growth inhibition. Such changes were observed both in ER-positive and ER-negative cells, indicative of the dual use of such a combination in prevention and therapy.

### 3.5. Curcumin and Sulfinosine (SF) (See [67])

The ineffectiveness of certain drugs in prolonged chemotherapy stems from the resistance that some cancers develop with time. This is one of the major obstacles in cancer therapy, especially in cancers that are multidrug resistant (MDR). The problem in finding a suitable cure for non-small-cell lung cancers is the MDR phenotype it exhibits. Treating MDR cells such as non-small-cell lung carcinoma NCI-H460/R cells with a commonly employed drug, SF (obtained by the amination and subsequent oxidation of 6-thioguanosine), in cell cultures has been shown to inhibit cell growth. This observed cytotoxicity is enhanced several folds when low doses of the natural compound, curcumin, are used in combination, which are otherwise ineffective unless very high concentrations are used. These compounds mediate a synergistic effect in regulating the cell cycle phases and downregulate MDR genes, thereby, enhancing tumor regression phenotypes even in the presence of mutated p53 molecules.

### 3.6. Curcumin and Celecoxib (See [68])

Cyclooxygenase-2 (COX-2) expression is central to the carcinogenesis of colorectal cancers. Compounds that regulate the expression or activity of COX-2 in cells may be instrumental in mediating chemotherapeutic effects on the tissue or cells. Celecoxib is a potent inhibitor of COX-2 and is presumed to target its active site. However, prolonged exposure to celecoxib results in cardiovascular problems. It appears that monotherapy regimes are very effective in inhibiting cancer growth, proliferation, metastasis, and invasion, as seen in numerous *in vitro *and *in vivo *models. However, prolonged exposures at concentrations relatively higher than what can be achieved with combination doses may result in unwanted side effects. Testing the efficacy of celecoxib with cucumin showed that at lower doses of celecoxib it was possible to enforce synergistic inhibitory growth effects on colon cells which expressed various levels of COX-2. Like many other *in vitro *investigations, this study emphasizes the fact that combining powerful drugs with naturally available potent compounds can reduce the dose needed to mediate potent anticarcinogenic effects with minimal side effects. Clinically, such studies are relevant as the doses used or needed are within the physiologically dose range. With colon cancer having such a high incidence rate in the western populations, such therapies can be taken as advantage, and biomolecules having preventive potential against the formation of precancerous lesions need to be supplemented in diets of patients at high-risk.

### 3.7. Coltect and 5-Aminosalicylic Acid (5-ASA) (See [69])

Coltect is a novel chemotherapeutic dietary drug with a formulation of curcumin, a turmeric extract (95% curcuminoids) mixed with turmeric powder 1 : 1, green tea (60% polyphenols and 25% EGCG) in a 2 : 1 ratio, and 0.1 mg/mL of L-selenomethionine. 5-ASA is an anti-inflammatory drug, which has been shown to have a preventive role in polyp formation that is thought to occur via the inflammation process in conditions like inflammatory bowl disorder. Coltect has been effective against HT-29 human colon adenocarcinoma grade II cells *in vitro,* and this nutraceutical complex in combination with 5-ASA has been shown to inhibit the formation or growth of chemically induced aberrant crypt foci (ACF) in rat models. The molecular mechanism by which this inhibition is mediated is via the inhibition of COX-2 pathways in HT-29 cells, which has been supported by *in vitro *studies of other groups [106, 107]. However, growth inhibition can be affected via COX-2-independent pathways possibly through mechanisms that are regulated by the functional polyphenol complex in coltect. Such complex mixtures are of clinical significance as many different control mechanisms can be regulated by the presence of individual constituents of the polyphenols which are a part of the formulated mixture of coltect.

### 3.8. Phenylethylisothiocynate (PEITC) and Curcumin (See [27])

Most prostate cancers begin as a hormone-dependent tumor, and the hormone is primarily androgen. However, the more aggressive forms of prostate cancer are androgen-independent and hormonal therapies fail to be effective. Alternate therapies are, therefore, necessary to treat such aggressive forms. Most cancerous cells express various surface receptors that propagate cellular growth. Targeting such receptors can be an effective chemotherapeutic approach. Curcumin, obtained from *Curcumin longa*, has been shown to inhibit the phosphorylation of EGFR, inhibit the Akt signaling pathway, and negatively regulate NF-*κ*B. It is an effective molecule against prostate cancer. Phenylethylisothiocyanate, a phytochemical in cruciferous vegetables, has been shown to inhibit prostate cancer cell growth *in vitro *and this observation has been supported by epidemiological studies showing that consumption of cruciferous vegetables has an inverse effect on prostate cancer risk. When two bioactive molecules with similar effects are used in treating hormonally independent tumors in affecting receptor mediated signaling, the effects are more pronounced than when used as individual compounds. With PEITC and curcumin, the observed effect was more additive than synergistic, but cell growth inhibition was profoundly affected by the inhibition of NF-*κ*B pathways and Akt signaling pathways. Such responses were seen at lower physiological achievable doses. These results were corroborated by *in vivo *studies in mice using human PC-3 prostate cancer cells [70]. Since EGCG has similar effects on prostate cancer cells, EGCG could also possibly serve as a substitute in place of curcumin for such a treatment strategy.

## 4. D-Limonene and Its Combination Therapies (See Table 3)

Although a few studies have shown that D-limonene, an abundant monoterpene in citrus oils, exhibits antimitogenic activity, its alcohol-derivated perillyl alchohol (PA) has a greater inhibitory effect on cell migration in cancerous cells [108]. A study by Reddy et al. [108] used subtoxic doses of PA to determine this effect. Further preclinical studies are necessary to determine the effective yet nontoxic serum/tissue concentration that can be achieved from a diet rich in citrus intake, in conjunction with phytonutrients of the same class or a different class. Not much is known about the percentage composition of D-limonene and its metabolized constituents that are required to achieve an effective monterpene anticarcinogenic activity. In comparison to its oxygenated derivatives, limonene has the least cytotoxic effect on both noncancerous and cancerous breast cell lines and, therefore, can be applicable in chemoprevention [109].

 D-limonene appears to be more effective against chemically induced colonic crypt foci [110]. These foci are preneoplastic lesions and are biomarkers for the progression into colon cancer. In colonic crypts that are chemically induced, limonene asserts its effect by inhibiting the activity of ornithine decarboxylase, an enzyme essential for the polyamine biosynthesis pathway. This pathway regulates the cell cycle, and D-limonene-dependent inhibition of ornithine decarboxylase (ODC) encourages an antiproliferative activity. If aberrant crypt foci are the initial markers for colon carcinogenesis, and D-Limonene and its derivatives assert their roles against initiation and promotion phases of cancer, then a diet rich in citrus foods can prevent crypt formation. Therefore, D-limonene appears to have potential as a chemopreventive agent in colon carcinogenesis. However, *in vivo *studies often do not correlate with results *in vitro *for many of the reasons discussed earlier. Once the intake of a compound is deemed safe for human consumption, it is imperative to analyze and study the mechanistic and metabolic functions in human subjects to determine the efficacy of the nutrient in question. As in the case of understanding limonene protection against colonic carcinogenesis, the studies were performed on rats and for shorter exposure time to the compound or its derivatives. Therefore, further *in vivo *models are required to determine the toxicity of the treatment for longer periods of time, as D-limonene is nontoxic but its alcohol derivatives could be toxic. 

### 4.1. D-Limonene and Docetaxcel (See [72])

Many combination studies are underway to determine an effective approach in treating advanced and aggressive prostate cancers. Docetaxel, a synthetic derivative of taxol, is primarily used to inhibit the microtubular structures in cancerous cells that support cell division. In addition to its role as a microtubule disruptive molecule, it has a host of inhibitory actions on genes which regulate cell proliferation, mitotic spindle formation, transcription factors, and oncogenesis. It also upregulates genes involved in apoptosis and cell cycle progression in prostate cancer. D-Limonene, discussed earlier has been shown to have anti-prostate carcinogenic effects at low dose concentrations. Logically; therefore, combining the two compounds may have a plethora of positive antitumor functionalities. In a study by Rabi and Bishayee [72], the combined treatment enhanced the sensitivity of DU145 prostate cancer cells that are known to be apoptotic resistant. This enhanced sensitivity was thought to be mediated by reactive oxygen species (ROS) generation and activation of caspase 3 and 9. Such a positive *in vitro *outcome warrants further investigations *in vivo*, in models that mimic the progression of the disease, before it can be used in dietary supplements for therapy.

### 4.2. Lycopene and Fru/His (See [74, 111–114])

Serum lycopene (a carotenoid) levels have been shown to have an inverse correlation with prostate cancer risk. A diet-based population study showed that, of all the carotenoids assessed, high serum lycopene levels showed a statistically significant lower prostate cancer risk. Further analysis of their data revealed that lower serum lycopene levels in conjunction with *β*-carotene supplements were effective against lowering the risk of prostate cancer, suggestive for a combinatorial therapy [111]. Certain dietary compounds can be the source of cancer formation as seen with prostate cancer. It is believed that the nonfat portion of milk and excess calcium are some main factors in prostate cancer risk [112]. Numerous *in vitro *studies have shown that carotenoids have a greater influence in reducing tumors of the prostate origin, and lycopene and 1,2-dihydroxyvitamin D3 are at the forefront as risk reduction factors. In addition to their role as potent inhibitors of prostate cancer growth, they are biologically safe and cheaper forms of treatment. 1,2-dihydroxyvitamin D3 and lycopene have physiologically different roles, but combined they modulate pathways to synergistically inhibit proliferation and differentiation at much lower concentrations [113] and bear additive effects on cell cycle progression. 

The assessments that lycopene is a safe dietary molecule with anticancer properties is supported by a number of population epidemiological and cohort-based studies [112]. However, it is important to ensure that the statistical models used are able to adjust for many parameters for a true significant outcome. Regardless of the statistical model employed in these assessment studies, lycopene has emerged as a potent risk-reducing factor of prostate cancer and has been even supported by a study that was carried out across 28 countries. Intervention combination studies have not yet been performed. However, *in vivo-*based studies in mice models have shown that lycopene administered in the form of tomato powder and broccoli powder in a 10 : 10 ratio, increases its serum concentration to about 538 nM/g with about 0.4 nM/g concentrated in the prostate tissue itself. Diet-based intervention studies are required to determine the formulated diet required to improve the availability in the serum of patients and enhance the localized concentration of the molecule in the tissue. Such a diet-based treatment may serve as a suitable chemopreventive approach against prostate cancer or with patients at high-risk of the disease. Even though bioactive molecules successfully work in administering their protective functions *in vitro*, it appears through *in vivo *studies that diet and availability crucially dictate outcomes. A critical question to be asked is what factors constitute a perfect blend of bioactive mixtures. With the current research thus far, it is hard to address what the cut-off ratios are that need to be used in a diet that contain mixtures of potent nutraceuticals to coordinate similar effects clinically. Possibly a slight change in concentration of even one of the effective biomolecules may render the mixture ineffective in its function. It is rather an important task for pharmaceutical chemists and nutritionists to determine the ratios of effective biomolecules in a mixture and determine the pharmacokinetics and dynamics of that mixture. 

Fru/His, a ketosamine, is also a derived product from tomatoes obtained by the reaction of a carbohydrate with an amino acid. This particular ketosamine has been found to assert chemopreventive effects by synergistically enhancing the activity of lycopene, by neutralizing ROS species and inhibiting DNA damage. Therefore, the complex of these two molecules may have a pivotal role in prostate cancer prevention. Although a rat model was used to determine the results of the treatment and pharmacokinetics of the compound are still not known, the combination of the two seemed to preferentially localize in the prostate more than in other tissues that were tested [74]. 

Occasionally, a combination may fail to incite anticarcinogenic effects as was seen by Mossine et al. [114]. Their experiments were conducted on the prostate adenocarcinoma rat model that was used by other groups, and their data had contradictory results to the effective action of lycopene itself and in conjunction with other micronutrients. Their study revealed that lycopene was not able to inhibit or reduce tumor load alone or in combination and that selenium alone in the mixture was able to induce antitumorigenic effects. Such outcomes are important as they open up more questions as to why a molecule that affects a given pathway behaves differently when tested within the same experimental model. Is it always dose or concentration or does molecule preparation and delivery impart effects on the efficacy of a drug?

### 4.3. Lycopene and Docetaxel (See [75])

Docetaxel is a potent chemotherapeutic drug that is clinically used to treat patients with advanced metastatic prostate cancers. Although the drug extends survival, it is for a very limited time period and with a poor prognosis. Lycopene, a natural compound, has been shown to have strong cancer inhibitory properties against the prostate tissue. One study tested the possibility to use this combination of compounds to enhance survival of patients that were detected with aggressive, androgen-independent tumors. As predicted, docetaxel inhibited tumor growth in nude mice that bore tumor xenografts of human DU 145 cells. Analysis of molecular mechanisms revealed that the action of docetaxel was on regulating the insulin-like growth factor receptor (IGFR) pathway by suppressing IGF, and this effect was synergistically enhanced in the presence of lycopene. Together the molecules asserted negative downstream effects on Akt signaling pathways and suppressed survivin, products of which have been known to maintain tumor growth and enhance metastasis. Clinical trials using this combination may prove effective in treating patients that express high levels of IGFR in the prostate tumor and extend survival for a longer duration than what is possibly achieved by docetaxel alone, which is about 18–20 months.

## 5. EGCG and Quercetin (See [31])

EGCG exhibits strong chemopreventive and therapeutic activities as it influences many pathways as shown in Figure 1. Some of the mechanistic pathways are involved in regulating the levels of Bcl2, survivin, and XIAP and activation of caspase-3/7 to induce apoptosis. EGCG is also involved in inhibiting genes that are required for transition from epithelial to mesenchymal cells and retards migration and invasion which are primarily advantageous in terms of controlling aggressive tumors. EGCG mediates such synergistic actions in conjunction with quercetin to retard the self-renewal properties of cancer stem cells (CSCs), a population that, if inhibited, can influence tumor regression. Quercetin, a polyphenol, downregulates the expression of the heat shock protein (Hsp90) known to influence apoptosis and growth inhibition of prostate tumors. Therefore, the combination of these molecules modulates their respective therapeutic effects to mediate synergistic growth retardation of CSCs. The study by Tang et al. [31] used relatively higher concentrations of EGCG (60 *μ*M) in the presence of 20 *μ*M quercetin. Probably concentrations of EGCG that can mediate similar synergistic levels, albeit at lower doses, need to be investigated, and the therapeutic potential across cancer stems cells of other origins need to be assessed if clinical applications are to be considered.

## 6. Resveratrol and Estrogen (See [39])

Selective estrogen receptor modulators that are used in the clinical treatment of breast cancers display dual agonist/antagonist effects in the tissues, especially in cancer initiation and progression. Drugs like tamoxifen emulate antagonistic effects on estrogen to contain the tumor. Agonistic-estrogen-like activity can in some instances enhance tumor progression which is not desired in most clinical treatments. Resveratrol, a polyphenolic compound abundant in grape skin and grape products including wine, is known to have chemopreventive properties as supported by numerous *in vitro *and *in vivo *studies. However, based on the experimental cell type, resveratrol induces either agonistic or antagonistic effects that can be weak or very pronounced. Resveratrol agonistic effects are totally reversed in the presence of estrogen, possibly mediated through estrogen receptor *β*. This reversal of effects is pertinent to prevention of breast cancer lesions in ducts that could become long-term neoplastic and cancerous. Of its many cancer protective functions, resveratrol in combination with glucan are potent immunomodulators by upregulating Cdc42 expression [115]. When natural compounds exhibit multi-chemopreventive properties, conjugation therapies are advantageous over monotherapies. Albeit not clinically tested, harnessing cancer preventive and immune modulating functions of nutraceuticals seems to be a plausible approach to targeting hormonally independent aggressive tumors. 

### 6.1. Resveratrol, Quercetin and Catechin (See Table 4 and [77])

The protective functions of polyphenols are manyfold. Numerous studies have analyzed their protective and therapeutic functions *in vitro *on tumor initiation that was chemically induced or *in vivo *via cellular implanted tumor formation. Few studies have established the functions of combined polyphenols on established tumors, as the majority of investigations have focused on individual mechanistic effects of the compounds. Dietary serum concentrations are influenced by the individual percentage of biomolecules present in the diet. Therefore, individual protective assessments of a compound show higher dose requirements, whilst mixtures may require lower doses to achieve the same effects. Additive and synergistic effects of compounds occur if their individual functions are enhanced in the presence of other molecules, perhaps by reinforcing the serum stability and availability of the various compounds in the mixture. Such observations were seen in both *in vitro *and *in vivo *testing of a mixture of three polyphenols, resveratrol, quercetin, and catechin, albeit pharmacokinetics studies are warranted.

### 6.2. Resveratrol and Cyclophosphamide (See [78])

Cyclophosphamide, a neoplastic drug, has a broad spectrum of activity on a variety of cancers, including breast cancers. The shortfall of the drug is its myriad of toxic effects on other systems. Dose reduction of the compound would be a means of reducing its toxicity without compensating its anticarcinogenic activity. Resveratrol has been shown to successfully lower the effective dose of cyclophosphamide without altering its anticarcinogenic activity. Both of the compounds together synergistically enhance caspase-mediated cytotoxic activity, as demonstrated in MCF-7 cells, an aggressive breast cancer cell line (Table 4). The combination therapy resulted in the upregulation of p53, proapoptotic genes, Bax and Fas, and downregulation of antiapoptotic gene Bcl-2, suggestive of an apoptotic mechanism involved in cell death.

### 6.3. Resveratrol and n-Butyrate (See [79])

n-Butyrate is a short chain fatty acid produced by bacterial fermentation of fiber in the colon. The compound is a known differentiating agent and induces an epithelial phenotype in certain cultured cells. n-butyrate is a potent histone deacetylase (HDAC) inhibitor as well and one of its differentiation-inducing properties stems from its ability to inhibit HDACs. Resveratrol, discussed above, induces apoptosis through other mechanistic pathways. The combination of two bioactive molecules influencing apoptosis via different mechanistic pathways may associate to render an apoptotic phenotype in cancerous cells and inhibit tumor formation and progression. The 2 mM dose of n-butyrate used in the Wolter and Stein study [79] is probably much higher than what can be physiologically achieved. This dose is probably suitable for treatment of colon cancers where higher molar doses of n-butyrate are possible. However, n-butyrate is highly unstable, and its serum concentrations are lower than 2 mM. Since this molecule is a differentiating agent, its clinical use in treatment of other cancers is relevant. However, such therapies require absolute lower effective doses and can probably be achieved by combining with molecules other than resveratrol or modifying the compound to specific conjugates to reach serum concentration levels.

### 6.4. Resveratrol and 5-Fluorouracil (5-FU) (See [80])

5-fluorouracil inhibits thymidylate synthase, prevents DNA proliferation, and induces DNA damage-related apoptosis in colon cancer cells. Phase I clinical trials using a combination of resveratrol and grape powder have shown that resveratrol at low doses inhibit *Wnt*, a gene that is upregulated in colon cancers. Taking advantage of therapeutic effects of nutraceuticals, combined therapy of aforementioned resveratrol with 5-FU surfaces as a principal strategy in treating colon cancers. When used in combination, the presumption is that either additive or synergistic effects of the two could mediate tumor inhibition by modulating their individual apoptotic effects. The concern in using resveratrol is that higher concentrations of the doses are required in the treatment which clinically may not be reached through dietary consumption.

### 6.5. Genistein and Resveratrol (See [81])

Genistein and resveratrol as individual phytochemicals are very effective in the treatment and prevention of prostate cancer progression in rodent studies. Poorly differentiated prostate cancers often fail to respond to androgen-dependent treatments, and alternate treatments are required. Androgen receptors likewise have two functional roles, one as a tumor suppressor in normal prostate tissue and the other as an oncogene in neoplastic transformation, where it is altered either by mutations or DNA damage. Genistein and resveratrol used in an *in vivo *rat-based study, modeled to understand the mechanistic action of combined treatments in the progression of prostate cancer, showed that they had more pronounced effects, albeit not synergistic. The statistically significant additive functions of reducing cell proliferation through mechanisms that regulate the androgen receptor levels and IGF-1, a biomarker found in patient serums with progressive and aggressive prostate cancers were achieved in combined therapies over the monotherapy regimes. Interestingly, the combination of genistein and resveratrol increased serum availability of both, but higher concentrations of resveratrol were achievable as compared to the single-dose regimen. Perhaps, absorption and stability of resveratrol were profoundly affected in a combined environment, which is clinically a clear advantage. The doses used in the study are physiologically safe and achievable *in vivo *by consumption of a soy-based diet high in the percentage of genistein. However, resveratrol is found in low levels in grape-based dietary products, and, therefore; a pure supplement of the compound is necessary in case that higher doses are required.

## 7. Genistein and Sulforaphane (SFN) (See [82])

Previous studies have shown that EGCG, a major polyphenol in green tea, can inhibit tumor growth through mechanisms that alter DNA methylation activity, reversing the expression of silenced genes involved in tumor inhibition in cancer cells. Hypomethylation of the promoters that are CpG-rich is more likely to be transcribed, with an exception of few like *hTERT*, the regulatory gene of telomerase [82, 116–119]. Epigenetics is a mechanism that has been studied for decades, and factors that regulate epigenetics are now believed to be very important as treatment possibilities in controlling tumors. DNA methylation and histone deacetylation are well known epigenetic mechanisms that regulate many of the genes involved in cancers of various origins. Genistein combined with SFN, an histone deacetylase inhibitor (HDACI) has been successful in inducing the transcription of genes involved in regulating cell cycle by reversing the hypermethylated states of their promoters. This change was observed at low doses and was enhanced in the presence of sulforaphane more than that when genistein was used alone. However, *in vivo *studies of the same are warranted to determine epigenetic behavior of the dietary compounds before applications to human treatments are considered. 

### 7.1. Benzylisothiocynate (BITC) and SFN (See [83])

BITC and sulforaphane are ITCs derived from cruciferous vegetables like broccoli. Individually both these molecules exert potent chemotherapeutic properties strongly supported by numerous studies. Oddly, even though both are isothiocyanates, they exert their therapeutic effects by controlling different pathways involved in tumorigenic inhibition. STAT3, a member of the STAT group of transcriptional factors, is required for early development and is dispensable in adult tissues. However, there appears to be a correlation between the constitutive expression of STAT3 and tumor development, indicative of its role as an oncogene. This gene appears to have important roles in cell proliferation, angiogenesis, and metastsis, a crucial requirement of tumor survival. Both BITC and sulforaphane have cancer inhibitory effects, affecting independent cell signaling pathways. However, the sequential combination of the two has been shown to regulate the STAT3 gene and others (Table 4), thereby, inducing apoptosis. How dietary molecules are presented to the cells *in vitro *is important to its cellular mechanistic actions. In the study by Hutzen et al. [83], sequential addition of BITC to the cells after sulforaphane treatment was performed, which enhanced the reduction of STAT3 levels; however, simultaneous additions were not performed. Simultaneous additions would be important for any combination study to determine possible synergistic, additive, or antagonistic effects between the compounds. Preclinical studies should include various combinatorial interactions of the nutraceuticals being tested to determine the best way of using combined molecules in therapy.

### 7.2. SFN and Apigenin (See [84])

Phase I and Phase II enzymes are extremely important to cancer prevention. Dietary foods are sometimes modified to produce carcinogens through metabolism by the action of Phase I enzymes. Subsequently, the action of Phase II enzymes rapidly metabolizes these products to more soluble forms that are eliminated as body waste. Phase II enzymes are more concentrated in the duodenum and small intestine and less available in the colon. Increasing the availability of these enzymes in the colon can get rid of harmful carcinogens reducing the incidence of colon cancers, and, therefore, dietary supplements that induce Phase II enzymes would be promising tools for colon cancer prevention. SFN, an isothiocyanate, and apigenin, a flavanol, have independent cancer preventive functions. SFN is a strong inducer of UDP-27 glucuronyltransferase (UGT1A1). UGT1A1 is a major player in the detoxification process of carcinogens formed in the body and, therefore, is a potent Phase II enzyme. Treating nondifferentiated colon cells with a combination of SFN and apigenin was found to synergistically induce the expression of UGT1A1 suggesting a possible dietary tool for colon cancer prevention. The *in vitro *dose of the individual compounds used in the study was at physiological safe levels and can be easily achieved *in vivo.*

### 7.3. SFN and 3,3′-Diindolylmethane (DIM) (See [85])

The importance of investigating the roles of dose combinations on chemopreventive or therapeutic functions has been well dissected in a study by Pappa et al. [85]. Lower doses of SFN demonstrate antagonistic effects on cell proliferation and higher doses of both compounds had synergistic effects. Synergism of compounds is preferred if the outcome is tumor regression, but in clinical treatments synergistic actions should be mediated at safe lower concentrations rather than at cytotoxic levels. Presumably, the choice of compounds used, based on the genetic or cellular function required, is imperative to the success of the treatment. Possibly, SFN can synergistically inhibit the proliferation of cancer cells with compounds other than DIM at much lower doses, which has been investigated in studies using SFN with flavanols. This clearly highlights the problems of using combined therapies, especially since dosage is of critical importance for the success of clinical trials.

### 7.4. SFN and Dibenzoylmethane (DMB) (See [28])

When seeking for dietary molecules with potential chemoprotective and therapeutic properties, it is essential to understand how they mediate their combined action. Based on mechanistic studies, only compounds that are able to achieve synergistic or additive inhibitory or inductive actions on cellular genes, pathways, and/or phenotypes can then be chosen for treatment, even though their individual actions may be more pronounced. DMB is antimutagenic. Patients with aberrant polyp crypt (Apc) mutations are prone to spontaneously form aberrant polyps in their intestinal tissue, which later can transform to colorectal cancers. Treatment with DMB found in licorice can effectively inhibit such mutations in Apc, thereby protecting individuals from aberrant polyp formations. This molecule, therefore, has potential in terms of colon cancer prevention. 

SFN has a myriad of chemopreventive functions as seen before in other studies and in various tissues. A combination of these two chemopreventive agents will have a profound impact on individuals that are at high-risk or reduce the incidence of colon cancers through dietary supplementation. The study by Shen et al. [28] showed that dietary intake of SFN and DMB negatively influenced the incidence and number of tumors formed in the Apc mice. The combined doses used were half that of the individual doses. However, the observed effects were still synergistic at these doses. Interestingly, the serum and plasma levels of SFN and DBM were lower in the combined doses than when the compounds were used individually. Regardless of the low serum availability, the combination was able to mediate synergistic tumor inhibitory effects. This has important clinical significance as it is possible to achieve greater tumor toxic effects at low plasma concentrations. Mechanisms that influence such actions at low serum availability need to be further investigated.

## 8. Future Directions

Chemopreventive agents are much sought after as an early interventional approach to prevent tumor development or to lower the incidence risk of cancers. Given that the current available methods of treatment are chemotherapy, radiation, and surgery, all of which can induce significant side effects, an urgent need for alternate or adjuvant therapies has arisen. Phytochemicals are relatively safe and abundantly available from dietary sources. Therefore, alternate medicine aims at harnessing the protective properties of these nonessential nutrients toward cancer prevention and treatment. A large database of studies supports the use of biomolecules in cancer treatment, albeit a majority of those are *in vitro *studies. Regardless of limited *in vivo *studies and clinical trials, phytochemicals show great promise in cancer treatment considering their safe use. Caution must be taken when addressing the efficacy of these molecules in clinical trials as many factors modulate their effects on cellular functions as detailed in Table 5. Combinatorial studies also show great promise, especially when lower nontoxic doses are required for prolonged periods to mediate potent chemotherapeutic functions with minimal side effects. Two of the major problems currently faced are dosage and delivery. To maintain a constant physiological serum dose availability, it is imperative that the agent is concentrated and stable in the tissue of concern. Combination technologies may be a solution to this problem. Nanotechnology is fast catching pace as the next level of technology in all spheres of science. Limited *in vitro *studies have shown that encapsulating dietary supplements in nanoparticles can effectively deliver the supplement and increase its stability and availability. Perhaps research needs to focus on such possibilities as avenues of using combination therapies.

## Figures and Tables

**Table 1:** Classification of nutrients as phytochemicals and their major food source availability.

Phytochemical class	Bioactive compound	Source	*Molecular formula	Reference
Alkaloid	Caffeine	Cacao, tea, coffee	C_8_H_10_N_4_O_2 _	[20]
	Theophylline	Cacao, tea, coffee	C_7_H_8_N_4_O_2_	

Monoterpenes	Limonene	Citrus oils from orange, lemon, mandarin, lime, and grapefruit	C_10_H_16_	[21]

Organosulfides	Allicin	Garlic	C_6_H_10_OS_2_	[22–25]
	Indole-3-carbinol	Cabbage	C_9_H_11_NO_2_	[26]
	Isothiocyanates	Broccoli	CNS	[27]
	Sulforaphane	Broccoli	C_6_H_11_NOS_2_	[28]

Carotenoids	Beta-Carotene, lycopene	Tomatoes	C_40_H_56_	[29]

Flavonoids	Epigallocatechin-3-gallate	Green tea	C_29_H_22_O_15 _	[30]
	Quercetin	Black tea	C_15_H_10_O_7 _	[31]
	Curcumin	Turmeric	C_21_H_20_O_6_	[32]

Phenolic Acids	Capsaicin	Chilli peppers	C_18_H_27_NO_3 _	[33]
	Ellagic acid	Black berries, raspberry	C_14_H_6_O_8 _	[34, 35]
	Gallic acid	Pomegranate, nuts	C_7_H_6_O_5_	[36, 37]

Stilbenes	Pterostilbene	Blueberries and grapes	C_16_H_16_O_3 _	[38]
	Resveratrol	Almonds, blueberries, grapes	C_14_H_12_O_3 _	[39]

Isoflavones	Daidzein	Soy	C_15_H_10_O_4 _	[9, 40]
	Genistein	Soy	C_15_H_10_O_5 _	

*Molecular formulas obtained through the PUBCHEM COMPOUND Database.

**Table 2:** Pharmacokinetic studies evaluating the bioavailability of phytochemicals at given doses.

Phyto chemical	Form	Dose	Model subject	Experimental setup	Maximum plasma concentration	Half-life (*h*)	Reference
Diadzein	Soy beverage	15 g Diadzein : genistein (9.27 : 10.51 mg)	Human postmenapausal women	Clinical	96.31 ng/mL	7.68	[40]
	Two soy capsules	Diadzein : genistein (7.79 : 22.57 mg)			96.02 ng/mL	6.67	

Genistein	Soy beverage	15 g Diadzein : genistein (9.27 : 10.51 mg)	Human postmenapausal women	Clinical	116.37 ng/mL	7.61	[40]
	Two soy capsules	Diadzein : genistein (7.79 : 22.57 mg)			216.84 ng/mL	7.96	

Curcumin glucoronide	Curcumoid powder form curcumin (75%), demethoxycurcumin (23%), and bisdemethoxy curcumin	10 g (*n* = 6)	Healthy human subjects (5 men and 7 women)	Clinical	2.04 ± 0.31	6.77 ± 0.83 for total curcumin conjugates	[41]
		12 g (*n* = 6)			1.40 ± 0.74		

Curcumin sulfate	Curcumoid powder form curcumin (75%), demethoxycurcumin (23%), and bisdemethoxy curcumin	10 g (*n* = 6)	Healthy human subjects (5 men and 7 women)	Clinical	1.06 ± 0.40	6.77 ± 0.83 for total curcumin conjugates	[41]
		12 g (*n* = 6)			0.87 ± 0.44		

Quercetin aglycone	Quercetin 500 plus capsule	500 mg of quercetin	Healthy human subjects (6 males and 4 female)	Clinical	15.4 ng/mL	3.47	[42]
Quercetin conjugates	Quercetin 500 plus capsule	500 mg of quercetin			336 ng/mL	Not given for plasma level, but renal clearance is 0.835	

Resveratol	Uncoated immediate-release caplets	500 mg resveratrol/caplet	Healthy human subjects	Phase I clinical test			[43, 44]
		0.5 g			72.6 (48.9)* ng/mL	2.85*	
		1.0 g			117.0 (73.1) ng/mL	8.87 (91.1)	
		2.5 g			268.0 (55.3) ng/mL	4.22 (51.6)	
		5.0 g			538.8 (72.5) ng/mL	8.52 (95.8)	

Sulforaphane	Broccoli raw	200 g	Healthy adult male subjects	Clinical	103 ± 31^@^, nM	3.8 ± 0.8^@^	[45]
	Broccoli cooked	200 g			31 ± 19^@^ nM	4.6 ± 0.8^@^	

EGCG	Beverage 200 mL	112 mg	Healthy human subjects	Clinical	Per dose (L^−1^) 0.51 × 10^−3^ ± 0.08 × 10^−3^	3.2 ± 2.1	[46]

D-Limonene oxygenated metabolite perillic acid	30–40 ounces of lemonade	447–596 mg D-limonene	Healthy human subjects	Clinical	2.08–13.98 *μ*M	12–24	[21]

Lycopene	Lycopene with up to 250 mL water	10–120 mg	Healthy adult male subjects	Clinical	Range between 4.03 and 11.27 *μ*g/dL (0.075–0.210 *μ*M)	Range between 28.1 61.6 h	[29]

*coefficient of variation; ^@^SD—standard deviation.

**Table 3:** Single-dose clinical studies evaluating the bioavailability of phytochemicals or their conjugated or active metabolites.

Phytochemical	Route of administration	Form	Bioavailability area under the curve (AUC)	AUC values	Reference
Diadzein	Oral	Soy beverage	107 ± 49.16 ng·h/mL	Adjusted to the dose	[40]

		Soy extract capsules	142.61 ± 43.94 ng·h/mL	Adjusted to the dose	[40]

Geistein	Oral	Soy beverage	121.48 ± 70.98 ng·h/mL	Adjusted to the dose	[40]

		Soy extract capsules	131.04 ± 60.79 ng·h/mL	Adjusted to the dose	[40]

Curcumin conjugates (glucoronide + sulfate)	Oral	Curcuminoid powder extract capsule form (10 g)	35.33 ± 3.78 *μ*g·h/mL	Relative	[41]
	Oral	Curcuminoid powder extract capsule form (12 g)	26.57 ± 2.97 *μ*g·h/mL	Relative	[41]

Quercetin aglycone	Oral	Capsule (500 mg)	62.5 ng·h/mL	Relative	[42]
Quercetin-conjugated metabolites	Oral	Capsule (500 mg)	2000 ng·h/mL	Relative	[42]

*Rersveratrol	Oral	Caplet ranging from		Relative for all	[43]
		0.5 g	223.7^*δ*^ ng·h/mL		
		1.0 g	544.8 (57.2) ng·h/mL		
		2.5 g	78.6 (36.2) ng·h/mL		
		5.0 g	1319 (59.1) ng·h/mL		

°Sulforaphane	Oral	200 g broccoli		Relative	[45]
		Raw	495 ± 40 nM·h		
		Cooked	286 ± 139 nM·h		

EGCG	Oral	Average 200 mL beverage	AUC^*κ*^	nd	[46]

D-Limonene (perillic acid a major active metabolite of d-Limonene)	Oral	40 oz of Lemonade	5.07 to 32.59 *μ*M·h	Relative	[62]

Lycopene	Oral	Liquid form (tomato paste)	(AUC)_0-96_	Relative	[29]
		10 mg	214 ± 124.8 *μ*g·h/dL		
		30 mg	416.4 ± 183.9 *μ*g·h/dL		
		60 mg	421.7 ± 59.3 *μ*g.h/dL		
		90 mg	598.9 ± 396.8 *μ*g·h/dL		
		120 mg	655 ± 298.6 *μ*g·h/dL		

^∗^AUC value measured for resveratrol was AUC infinity with the coefficient of variance denoted in the brackets against the mean value.

^*δ*^For the lowest dose of resveratrol AUC infinity value *n* = 1.

°AUC value measured for sulforaphane was AUC0-∞.

^*κ*^Based on the reference paper a list of various AUC values was given for different single doses as experimentally performed by different laboratories. Since the sample numbers were different, an average AUC value has not been given for this compound.

nd—not determined.

**Table 4:** Assessment of the chemotherapeutic and chemopreventive effects of nutraceuticals in combination studies.

Combination of nutraceutical	Dose used	Pathways affected or mechanistic action	Organ of study	Phase of study	Model of study	Reference
Curcumin + paclitaxel	50 *μ*M/L + 10–50 *μ*M/L based on the gene assessed	Inactivation of NF-*κ*B and other metastatic genes.	Breast	*In vitro *	Human breast cancer cells MDA-MB-435	[63]
Curcumin + paclitaxel	2% w/w 10 mg/kg	Inhibition of metastasis		*In vivo*	Human breast cancer xenograft model	

Curcumin + xanthorrhizol	Synergistic effect in the range from 5 to 20 *μ*g/mL	Induction of apoptosis	Breast	*In vitro*	Human breast MDA-MB-231 cancer cells	[64]

Curcumin + docosahexenic acid	Ratio of DHA to CCM MCF-7 55 : 30 *μ*M MCF10A 95 : 45 *μ*M MDA-MB 35 : 35 *μ*M SK-BR-3 60 : 40 *μ*M MDA-MB 50 : 25 *μ*M	Inhibition of proliferation, more synergistic in one of the 5 cell lines tested. Enhanced uptake of curcumin by the cells. Upregulated genes involved in cell cycle arrest, apoptosis, inhibition of metastasis, and cell adhesion. Downregulated genes involved in metstasis and invasion.	Breast	*In vitro*	Human breast cancer cells SK-BR-3, MDA-MB-231, MDA-MB-361, MCF-7, and MCF10AT	[65]

Curcumin + genistein	10 *μ*M + 25 *μ*M	Change in cell morphology and growth inhibition	Breast	*In vitro*	T47D and	[66]
	10 *μ*M +25 *μ*M				MCF-7	
	11 *μ*M + 25 *μ*M				MDA-MB-231	

Curcumin + sulphinosine	15 *μ*M + 10 *μ*M	Alter multidrug resistance genes.	Lung	*In vitro*	NCI-H460/R	[67]
		Alters the cell cycle with cells inhibited primarily in the S G2/M phase of the cycle				

Curcumin + celecoxib	10–15 *μ*M/L + 5 *μ*M/L	Inhibition of cell proliferation and induction of apoptosis.	Colon	*In vitro*	HT-29	[68]
		Possible inhibition of Cox-2 pathways or through non-Cox-2 pathways			IEC-18-K-ras (Cox-2, high levels) Caco-2 (COX-2, low levels), and SW-480 (no COX-2)	

Coltect + 5-aminosalicylic acid (ASA)	Coltect only 20 *μ*M	Inhibition of tumor growth by induction of apoptosis.	Colon	*In vitro *	HT-29 cells	[69]
	150 mg/kg + 50 mg/kg	Inhibits abnormal crypt formation		* In vivo*	Chemical induction of tumors by 1,2-dimethyl hydrazine (DMH) model in rats.	

Curcumin + PEITC	25 *μ*M + 10 *μ*M	Additive effectives in the induction of apoptosis.	Prostate	*In vitro *	PC-3 C4 cell line	[27]
	3 *μ*M + 2.5 *μ*M	Inhibition of tumor growth through inhibition of Akt and NF-*κ*B pathways.		* In vivo*	NCr-immunodeficient (nu/nu) mice bearing s.c. xenografts of PC-3 human prostate cancer cells	[70]

Pure 3—curcumin + resveratrol + EGCG;	Individual compounds, Percentage composition in the diet not defined	Inhibit growth by inhibiting hedgehog signaling pathways.	Prostate	*In vitro *	PC-3, LnCaP and mouse cell line TRAMP-C2	[71]
Pure 4—apigenin + baicalein + genistein + quercetin; Pure 7—Pure 3 + Pure 4; Crude 7—soy + sencha leaves + turmeric + yucca roots + saw palmetto + chamomile flowers + gingko		Reduce or delay the onset of tumors.		*In vivo*	Transgenic adenocarcinoma of the mouse prostate (TRAMP) mice	

D-Limonene + docetaxcel	0.2 mM + 1.9 nM	Induction of apoptosis by the regulation of proteins involved in mitochondrial apoptotic pathways	Prostate	*In vitro *	Human prostate carcinoma DU-145 and normal prostate epithelial PZ-HPV-7 cells	[72]

Tomato powder + broccoli powder (10 : 10) g/100 g of diet	11 nM of lycopene per g of diet and broccoli powder, 1.6 *μ*M of glucoraphanin, 5.9 *μ*M of glucobrassicin, 3.9 *μ*M of gluconasturtiin, and 2.1 *μ*M of neoglucobrassicin.	Reduction of tumor growth mediated by reduced cell proliferation and induction of apoptosis	Prostate	*In vivo *	Dunning R3327-H prostate adenocarcinoma model	[73]

Lycopene + ketosamine (fructose/amino acid Fru/His)	1 *μ*M/L + 2 mM/L	Synergistic effect in inhibiting cell proliferation mediated processes. Antioxidant activity to prevent initiation of tumors.	Prostate	*In vitro *	Mat-Lylu rat cells	[74]
	20 *μ*M/L + 5.6 mM/L	Reduce tumor growth and volume.		* In vivo *	Subcutaneous injections of Mat-Lylu cells in male Copenhagen rats	

Lycopene + docetaxel	1 *μ*M + 1 nM	Synergistically enhances the antiproliferative effects of docetaxel.	Prostate	*In vitro *	Human PC-3, LnCaP, DU145 cells	[75]
	15 mg/kg lycopene + 10 mg/kg docetaxel	Reduced tumor volume and growth by affecting the levels of IG-FR receptor that is highly expressed in a majority of prostate tumors. Inhibited Akt signaling and suppressed surviving necessary for tumor growth		*In vivo *	Xenograft of DU145 cells in NCR-nu/nu (nude) mice	

Quercetin chalcone (QC) and a pH-modified citrus pectin (MCP)	1.6 mg/mL + 1.6 mg/mL	Reduction in the growth of solid primary tumors	Colon	*In vivo*	Balb/c mice	[76]

Quercetin + EGCG	20 *μ*M + 0–60 *μ*M	Inhibits the self renewal capacity of prostate cancer stem cells (PCSCs) by synergistically inducing apoptosis decreasing cell viability in spheroids, cell migration, invasion and colony formation	Prostate	*In vitro*	Prostate cancer stem cells (PCSCs)	[31]

Resveratrol + estrogen (E2)	10 *μ*M + 1 nM	Antagonistic estrogenic effects in suppression of progesterone receptor	Breast	*In vitro*	Human MCF-7 cells	[39]

Resveratrol + quercetin + catechin	Either all at 0.5 *μ*M and 5 *μ*M, or 20 *μ*M	Synergistically inhibited cell proliferation and induced apoptosis.	Breast	*In vitro *	Human MDA-MB-231 cells	[77]
		Inhibited cell cycle progression with predominat cell cycle arrest in the G2 phase				
	0.5, 5, and 25 mg/kg body weight in a 100-*μ*L volume	Reduced primary tumor growth and, therefore, inhibit tumor progression		*In vivo*	Breast cancer xenografts in mouse models	

Resveratrol + cyclophosphamide	50 *μ*M + 5 mM	Inhibit cell proliferation via capase mediated cytotoxicity. Enhanced proapoptotic genes Bax, Fas and suppressed anti apoptotic gene Bcl-2	Breast	*In vitro*	MCF-7	[78]

Resveratrol + n-Butyrate	50 *μ*M + 2 mM/L	Inhibited cell proliferation and induced differentiation. Attentuated p27 (Kip1) levels but enhanced p21 (Waf1/Cip1) expression.	Colon	*In vitro*	Caco-2	[79]

Resveratrol + 5-Fluorouracil	200 *μ*M + IC_50_ 800 *μ*M	Inhibited cell proliferation and induced apoptosis by increase in capase 6 activity	Colon	*In vitro*	HCT116 p53+/+ and p53−/−	[80]

Resveratrol + genistein	250 mg/kg each in the AIN-76 diet	Suppressed prostate cancer development and mediated apoptosis by affecting the expression of steroid-receptor coactivor-3 and insulin-like growth factor-1	Prostate	*In vivo*	Simian Virus-40 T-antigen-(SV-40 Tag-) targeted probasin promoter rat model, a transgenic model of spontaneously developing prostate cancer.	[81]

Genistein + sulforaphane	5 *μ*M/L + 15 *μ*M/L	Affected DNA methyltransferase activity and reversed the gene expression of promoter hypermethylated genes of retinoic acid receptor h (RARb), RARB, p16INK4a p16 and O6-methylguanine methyltransferase enhanced growth inhibitory effects	Esophagous	*In vitro*	KYSE 510 cells	[82]

Sulforaphane + benzylisothio- cyanite	10 *μ*M + 10 *μ*M	Changed cell morphology and inhibited cell proliferation. Reduced cell viability that correlated with reduced pSTAT3 levels and an increase in PARP Cleavage	Pancreas	*In vitro*	PANC-1 cells	[83]

Sulforaphane + apigenin	10 *μ*M + 10 *μ*M	Synergistically induced phase II enzyme UDP-glucoronyl transferases (UGT1A1) transcript but to a lesser effect the protein level. Mediates this action by the induction of NF-*κ*B	Colon	*In vitro*	CaCo-2	[84]

Sulforaphane + 3,3′-diindolylmethane (DIM)	2.5 *μ*M + 20 *μ*M	Has an antagonistic effect at low concentration on cell growth.	Colon	*In vitro*	Human colon cancer 40–16 cell line randomly derived from HCT116 clone	[85]
	Total concentration 40 *μ*M	At cytotoxic concentrations of the compounds has synergistic effects on growth inhibition				

Sulforaphane + dibenzoymethane (DMB)	AIN-76A diet supplemented with 300 ppm SFN and 0.5% DMB	Blocked colon tumor development	Colon	*In vivo*	Male Apc/^min +^ mice	[28]

**Table 5:** Factors conducive to the anticarcinogenic efficacy of nutraceuticals.

Factors	Possible effects on the bioactive components in the dietary supplement
Bioavailability	Metabolism
	Time taken to achieve maximum plasma concentration
	Maximum plasma concentration, half-life

Method of ingestion	Oral
	Intraperitoneal
	Subcutaneous

Form of ingestion	Powder/capsule
	Liquid
	Cooked (solid)
	Raw (solid)

Formulation	Ratio of pure to the compound conjugates

Stability	Preference for an acidic or basic environment (pH)

Mechanism of action	Direct via receptors on the cell surface or into the nuclear region via channels
	Indirect conjugated metabolites affecting parts of metabolic pathways

**Table 6:** Surface receptors expressed by breast cancer cells that alter their sensitivity to treatment.

	Receptors on the surface			Cancer type	Phenotype	Reference
	ER	PR	Her2			
Breast cancer cell line						
SK-BR-3	Negative	Negative	Positive	Adenocarcinoma	Invasive	[65]
MDA-MB-231	Negative	Negative	negative	Adenocarcinoma	Invasive	[65]
MDA-MB-361	Positive	Negative	Positive	Adenocarcinoma	Metstasis	[65]
MCF-7	Positive	Positive	Negative	Adenocarcinoma	Invasive	[65]
MCF10AT	Positive	Isoform B of PR and not A	Variable	Premalignant model for cancer development	Premalignant	[65]
